# Foliar Application of Nano-Silicon Improves the Physiological and Biochemical Characteristics of ‘Kalamata’ Olive Subjected to Deficit Irrigation in a Semi-Arid Climate

**DOI:** 10.3390/plants11121561

**Published:** 2022-06-13

**Authors:** Islam F. Hassan, Rahaf Ajaj, Maybelle S. Gaballah, Chukwuma C. Ogbaga, Hazem M. Kalaji, Harlene M. Hatterman-Valenti, Shamel M. Alam-Eldein

**Affiliations:** 1 Water Relations and Field Irrigation Department, Agricultural and Biological Research Institute, National Research Center, Giza 12622, Egypt; msgaballa54@yahoo.com; 2 Department of Environmental and Public Health, College of Health Sciences, Abu Dhabi University, Abu Dhabi 59911, United Arab Emirates; rahaf.ajaj@adu.ac.ae; 3 Department of Microbiology and Biotechnology, Nile University of Nigeria, Abuja 900001, Nigeria; 4 Department of Plant Physiology, Institute of Biology, Warsaw University of Life Sciences-SGGW, 02-776 Warsaw, Poland; hazem_kalaji@sggw.edu.pl or; 5 Institute of Technology and Life Sciences, National Research Institute, Falenty, Al.Hrabska 3, 05-090 Raszyn, Poland; 6 Department of Plant Sciences, Department 7670, North Dakota State University, P.O. Box 6050, Fargo, ND 58108, USA; h.hatterman.valenti@ndsu.edu; 7 Department of Horticulture, Faculty of Agriculture, Tanta University, Tanta 31527, Egypt; shamel.alameldein@agr.tanta.edu.eg or

**Keywords:** abscisic acid, antioxidants, chloroplast degeneration, drought, malondialdehyde, nanoparticles, oxidative stress, photosynthesis

## Abstract

In Egypt’s arid and semi-arid lands where the main olive production zone is located, evapotranspiration is higher than rainfall during winter. Limited research has used nanomaterials, especially nano-silicon (nSi) to improve the growth, development, and productivity of drought-stressed fruit trees, amid the global water scarcity problem. To assess the role of nSi on drought-sensitive ‘Kalamata’ olive tree growth, and biochemical and physiological changes under drought conditions, a split-plot experiment was conducted in a randomized complete block design. The trees were foliar sprayed with nSi in the field using nine treatments (three replicates each) of 0, 150, and 200 mg·L^−1^ under different irrigation regimes (100, 90, and 80% irrigation water requirements ‘IWR’) during the 2020 and 2021 seasons. Drought negatively affected the trees, but both concentrations of nSi alleviated drought effects at reduced irrigation levels, compared to the non-stressed trees. Foliar spray of both concentrations of nSi at a moderate level (90% IWR) of drought resulted in improved yield and fruit weight and reduced fruit drop percentage, compared to 80% IWR. In addition, there were reduced levels of osmoprotectants such as proline, soluble sugars, and abscisic acid (ABA) with less membrane damage expressed as reduced levels of malondialdehyde (MDA), H_2_O_2_ and electrolyte leakage at 90% compared to 80% IWR. These results suggest that ‘Kalamata’ olive trees were severely stressed at 80% compared to 90% IWR, which was not surprising as it is classified as drought sensitive. Overall, the application of 200 mg·L^−1^ nSi was beneficial for the improvement of the mechanical resistance, growth, and productivity of moderately-stressed (90% IWR) ‘Kalamata’ olive trees under the Egyptian semi-arid conditions.

## 1. Introduction

The olive tree (*Olea Europaea* L.), family Oleaceae, is an evergreen tree and one of the oldest fruit trees believed to be indigenous to the entire Mediterranean Basin. Its cultivation was considerably developed in Syria and Palestine and spread to the island of Crete towards Egypt [1,2]. The olive tree is a symbol of honor and culture and has been used as a prize for champions at the Olympic games [1]. Its global cultivated area is about 12,763,184 ha with a total annual production of approximately 23,640,307 t and an average yield of 1.85 t·ha^−1^. In Egypt, olive cultivation increased during the past two decades and reached 100,826 ha with an annual production of about 932,927 t and an average yield of 9.25 t·ha^−1^ [2]. Egypt ranked eighth and third in global olive oil and table olive production, respectively. A total of 10% of the crop is planted for the purposes of producing olive oil, whilst 90% is for pickling [3]. The most important grown cultivars in Egypt are Toffahi, Aggizi, Picual, Manzanillo, Kroniaki, Coratina, Chemlali, and Kalamata.

As one of the most famous olive cultivars worldwide, ‘Kalamata’ olive was introduced to Egypt from Greece about three decades ago. The tree is drought-sensitive, cold intolerant, and fairly large, with distinguished large grey-green foliage, which grows to twice the size of other olive cultivars. The tree has inconspicuous white flowers that produce large deep reddish-purple fruit. The fruit is soft, almond-shaped, and slightly bitter in flavor. Fruit are hand-picked to avoid bruising, and they contain moderate oil levels, so growers produce them mainly for use as table olives (pickled) and some oil. The optimal harvest time is late fall, whilst other cultivars are usually picked earlier [4].

Egypt’s Mediterranean climate of hot dry summers and warm humid winters is well suited for olive cultivation. In general, olives are drought-tolerant plants and can withstand hot and dry conditions; however, total annual production has been negatively affected in the past few years amidst climate change-induced water shortage and soil salinity problems [5]. Water scarcity has become a recent problem in Egypt and may translate to a limiting factor for the overall fruit industry in the future, particularly with the increased human demand for freshwater, which creates competition with agricultural activities [6]. Water resources and rainfall (20–200 mm annually) are limited, and the Nile River is the most important water resource [7]. Under such conditions, there is a need to reduce agricultural water demand and increase the economic productivity of water for the future expansion of olive agriculture in the water-scarce Mediterranean area [8]. Improving on-farm management of agricultural water through the utilization of advanced irrigation technology, (e.g., deficit irrigation) and improved irrigation scheduling, offer the prospect of significant increases in water productivity [9,10,11,12,13]. Deficit irrigation is a strategy where the amount of applied water is less than the full water requirements of a crop, and the resulting stress has minimal effects on crop yield [14]. Deficit irrigation effectively reduced water requirements, enhanced plant water use efficiency [15], and improved fruit quality of various deciduous and evergreen fruit trees [16,17,18], including olives, depending on the phenological stage when water shortage was applied, drought severity, and the cultivar [19,20,21,22,23,24].

The scenario of deficit irrigation is still under research for a few olive cultivars, such as ‘Kalamata’, which is classified as a drought-sensitive cultivar. Generally, drought mainly impacts plant morphology, physiology, and biochemistry [25]. Under such conditions, xylem vessels become susceptible to embolism or dysfunction, leading to lower hydraulic conductance and carbon intake, which in turn affect plant growth characteristics and productivity [26]. Drought stress causes a reduction in root and vegetative growth, number of leaves per branch, leaf area, and leaf water content [27]. It also causes photoinhibition of photosystem II (PS II), limits electron transfer from PSII to photosystem I (PSI) and induces stomatal closure. These eventually decrease CO_2_ fixation in the chloroplast during the Calvin cycle [28,29,30]. Drought stress causes an increase in the formation of reactive oxygen species (ROS) negatively affecting plant metabolism through oxidative damage by lipids, proteins, and nucleic acids [31,32].

Various reports have shown that the foliar fertilization of micronutrients such as Zn, B, Cu, Mn, Si, Se, and Fe are effective and induce a very rapid plant response. Their application can improve the nutrient balance of a plant, resulting in increased fruit yield and quality, better disease resistance, and alleviation of the adverse effects of drought and salinity stress [33,34,35,36]. During the past three decades, many problems in different fields of science and industry have been resolved using nanotechnology. Materials that are smaller than 100 nm, at least in one dimension, are generally classified as nanomaterials [37]. Nanomaterials could be used for designing new fertilizers [38], to ensure the effective delivery of the required nutrients to the plant and a very rapid plant response [33] with only one-third of the required conventional counterparts added to the environment [39]. In the same context, it was reported that silicon is mostly toxic to plants in its bulk form, whereas silicon nanoparticles were beneficial for plants [40].

Silicon (Si) is the second most abundant element in the soil; however, it is not considered an essential element for plant growth, development, or productivity [41]. Recently, Si has gained global attention because it is safe for the environment, induces disease and pest resistance in plants, and can reduce doses of pesticides applied for plant protection [42]. Silicon is beneficial for alleviating the nutrient imbalance stress and improving the growth, development, and yield of various plants [43]. It improves organogenesis, embryogenesis, growth traits, and the morphological, anatomical, and physiological characteristics of leaves. It also enhances tolerance to chilling, freezing, salinity, and drought and protects cells against metal toxicity, oxidative stress, and phenolic browning [40,44,45]. Foliar application of Si is most powerful for plants under stressful conditions, such as salinity, drought, flood, heat, cold, and even biotic stress [46]. Silicon can potentially decrease the negative effect of oxidative stress and offer slight resistance to some abiotic and biotic plant stresses. A large number of genes are activated by stress, and several Si-produced proteins that participate in biochemical pathways lead to the enhancement of stress tolerance [47]. The application of nano-silicon (nSi) has been suggested to enhance plant tolerance to drought stress by reducing the production of reactive oxygen species (ROS) in barley, wheat, faba bean, feverfew, strawberry, Mahaleb cherry, and mango [34,48,49,50,51,52,53,54]. The primary mechanisms of Si-mediated abiotic stress reduction include plant antioxidant system activation, co-precipitation and immobilization of toxic metal ions in the growth medium, and metal absorption and separation within the plants [55,56,57,58,59,60,61,62,63,64,65,66,67,68].

Limited research findings have been reported on the effect of nanoparticles (NPs) on woody plants, especially fruit trees. Most reports focused on the role of NPs to alleviate the effect of stressful conditions on fruit tree seedlings, whilst some concentrated on using NPs to improve the growth, yield, and fruit quality of fruit trees growing under non-stressful conditions. To the best of the authors’ knowledge, this is considered to be one of few reports to cover both goals and the first on a drought-sensitive olive cultivar. The aim of this research was to estimate the role of nano-silicon in alleviating the drastic effects of water stress on a drought-sensitive ‘Kalamata’ olive tree grown under semi-arid conditions. The hypothesis was that nano-silicon may enhance the physiological and biochemical characteristics of stressed ‘Kalamata’ trees by improving the resistance mechanisms, growth, and productivity.

## 2. Results

### 2.1. Yield

**Figure 1:**
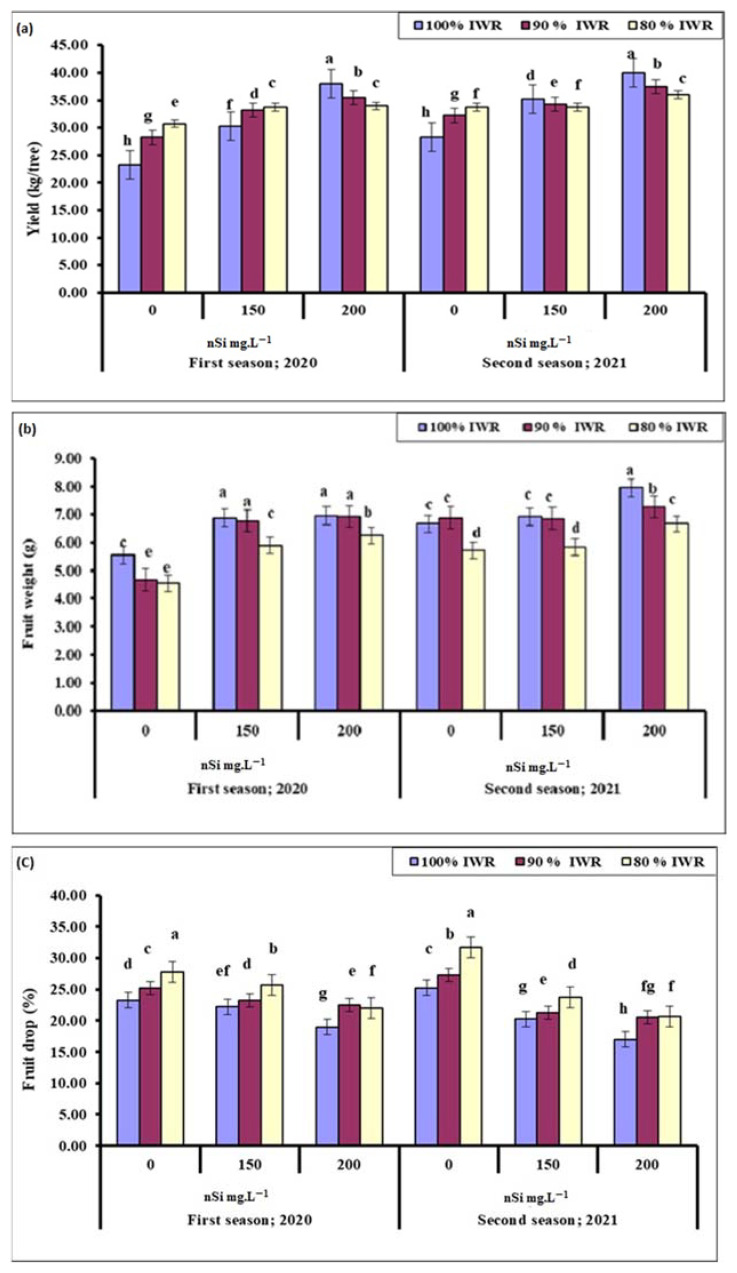
Effect of foliar sprayed nano-silicon (nSi) under different irrigation regimes on the yield (**a**), fruit weight (**b**) and fruit drop (**c**) of ‘Kalamata’ olive trees during the 2020 and 2021 seasons (*n* = 10). Means with similar letters for each season are not significantly different, using Duncan’s multiple range test (DMRT) at *p* ≤ 0.05. Error bars represent the standard error of the means.

Olive yield differed slightly from one season to another (Figure 1a). In the 2020 season, the yield per tree increased with increased nSi concentration, regardless of the water stress level, with the exception of trees that received 80% IWR. The highest yield per tree was recorded in trees sprayed with 200 mg·L^−1^ nSi at 100% IWR, while the lowest yield was recorded in the control trees. In the 2021 season, total yield increased with increased nSi concentration, regardless of the water stress level, with the exception of trees that received 80% IWR. Like the first season, the highest yield was recorded in trees sprayed with 200 mg·L^−1^ nSi at 100% IWR, and the lowest for the control. In addition, trees that received 200 mg·L^−1^ nSi at 80% IWR showed an improved yield when compared to those that received 150 mg·L^−1^ nSi at 80% IWR. Results suggest that nSi application increased ‘Kalamata’ olive yield when trees were stressed.

### 2.2. Fruit Weight

Unlike total yield, average fruit weight was the highest at 100% or 90% IWR with the application of either 150 or 200 mg·L^−1^ nSi (Figure 1b) during the 2020 season. Average fruit weight was the least at 80% IWR combined with either 150 or 200 mg·L^−1^ nSi; however, fruit were still heavier than those that were not sprayed with nSi, regardless of the water regime. During the 2021 season, the highest fruit weight was recorded at 100% IWR, combined with 200 mg·L^−1^ nSi. At the same concentration, fruit weight decreased as water stress severity increased. Similar fruit weight was noticed at 100% or 90% IWR in combination with either distilled water or 150 mg·L^−1^ nSi.

### 2.3. Fruit Drop

The more severe the drought condition was, the higher percentage the fruit drop was at either distilled water or 150 mg·L^−1^ nSi spray during the 2020 season (Figure 1c). The lowest fruit drop was noticed at 200 mg·L^−1^ nSi for non-stressed trees. Increasing the concentration of nSi to 200 mg·L^−1^ nSi under drought stress conditions (either 90% or 80% IWR) reduced fruit drop in comparison to the control. Increased fruit drop percentages with the application of 150 mg·L^−1^ at 80% IWR, compared to that at 90% IWR indicated that plant nSi was not that effective during the severe drought. However, the application of 200 mg·L^−1^ nSi was more effective at 80% IWR during the first season only.

### 2.4. Biochemical Characteristics

#### 2.4.1. Total Chlorophyll

**Figure 2:**
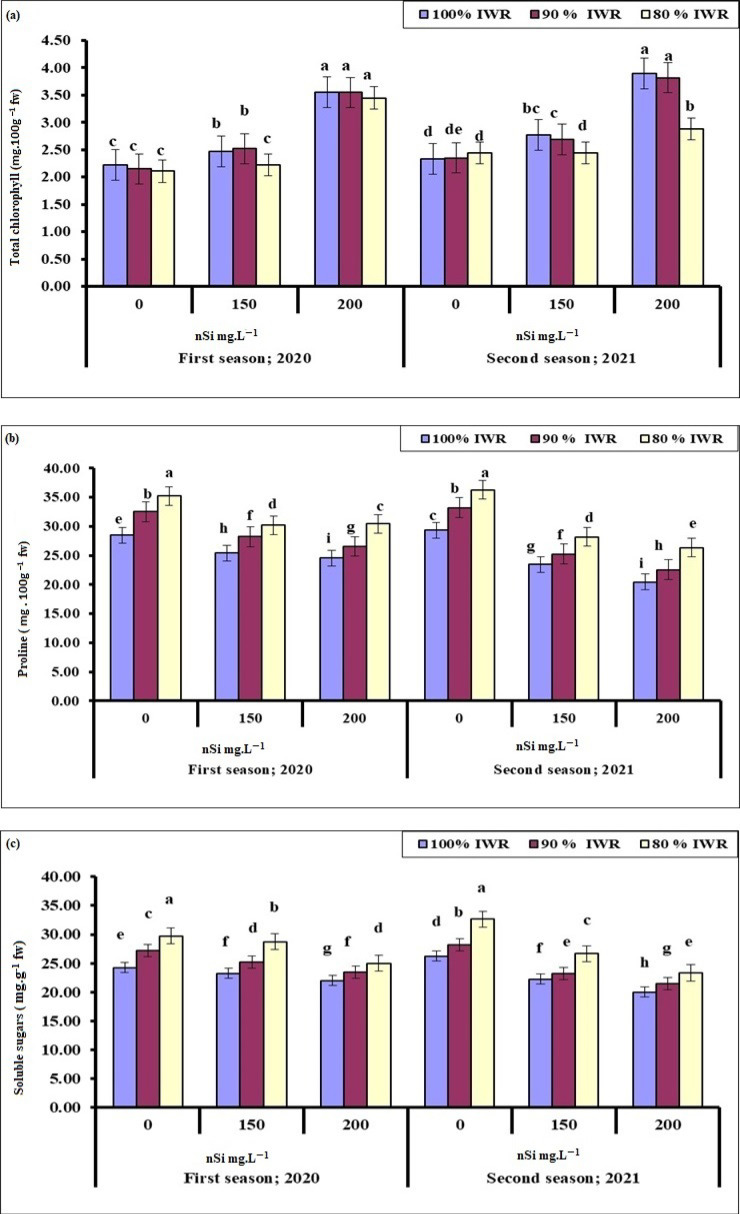
Effect of foliar sprayed nano-silicon (nSi) under different irrigation regimes on the leaf total chlorophyll (**a**), proline (**b**), and soluble sugars (**c**) of ‘Kalamata’ olive trees during the 2020 and 2021 seasons (*n* = 10). Means with similar letters for each season are not significantly different, using Duncan’s multiple range test (DMRT) at *p* ≤ 0.05. Error bars represent the standard error of the means.

Results indicated that generally, the higher the nSi concentration, the higher the leaf chlorophyll content was, regardless of the drought severity in both the 2020 and 2021 seasons. However, the difference between nSi (150 mg·L^−1^) at 90% IWR and the non-treated plants at any irrigation level was insignificant (Figure 2a). Similar results were noticed in both seasons; however, chlorophyll content was significantly decreased in trees received 200 mg·L^−1^ nSi at 80% IWR compared to the nonstressed ones during the second season.

#### 2.4.2. Proline

As osmoprotectant, proline increased with the increased levels of water stress. Unlike chlorophyll, leaf proline generally decreased with increased concentrations of nSi at all irrigation regimes during both seasons (Figure 2b). Trees subjected to 90% IWR and received nSi (200 mg·L^−1^) were less stressed and had lower proline compared to those that received 150 mg·L^−1^ in both seasons confirming the role of nSi in mitigating the drought effects and suggesting that irrigation at 80% IWR was really stressful for olive trees.

#### 2.4.3. Soluble Sugars

Like proline, leaf soluble sugars generally increased with increased levels of water stress, but they decreased with the increased concentrations of nSi in both seasons (Figure 2c). Trees sprayed with 200 mg·L^−1^ nSi at 80% IWR showed more soluble sugars than that of the control leaves during the first season, whereas they showed less soluble sugars during the second season, suggesting a cumulative effect of drought and nSi application from one season to another. nSi application mitigated the stress effect at 90%, compared to 80% IWR, suggesting that 80% IWR was really stressful to olive trees under the Egyptian semi-arid conditions.

### 2.5. Leaf Water Status and Membrane Damage Indicator

#### 2.5.1. Relative Water Content (RWC)

**Figure 3:**
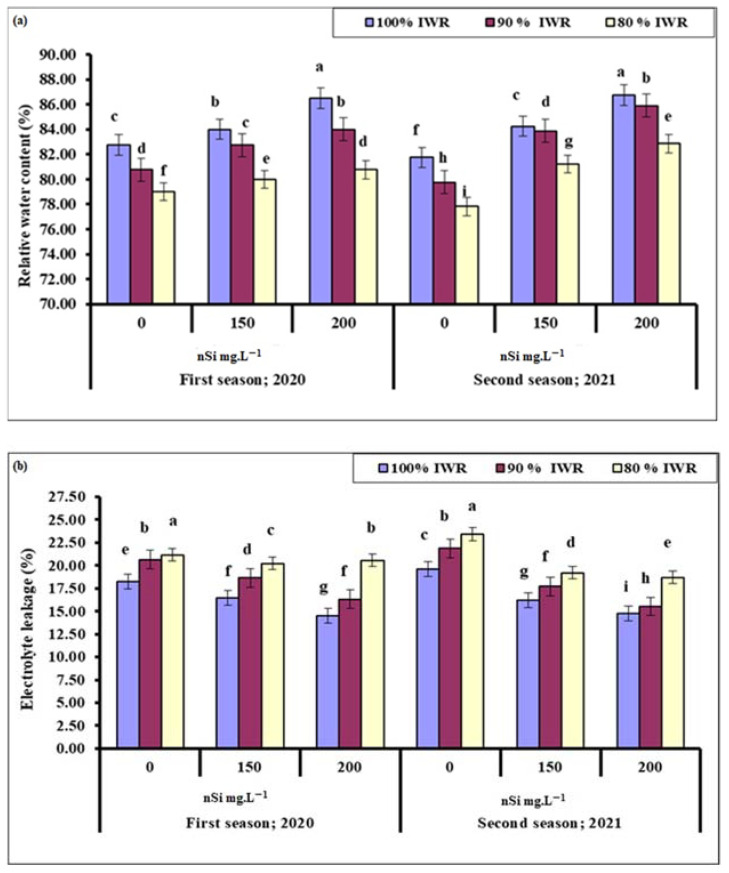
Effect of foliar sprayed nano-silicon (nSi) under different irrigation regimes on the leaf relative water content ‘RWC’ (**a**) and electrolyte leakage (**b**) of ‘Kalamata’ olive trees during the 2020 and 2021 seasons (*n* = 10). Means with similar letters for each season are not significantly different, using Duncan’s multiple range test (DMRT) at *p* ≤ 0.05. Error bars represent the standard error of the means.

The water contents of olive leaves were generally the highest in the control trees but decreased gradually with the severity of drought during both seasons. The application of nSi improved the RWC, which was the highest at 200 mg·L^−1^ (Figure 3a). The most pronounced effect was recorded for non-stressed trees that received nSi (200 mg·L^−1^) in both seasons. Relative water content (RWC) was negatively affected in response to nSi and water stress levels, in comparison to proline and soluble sugars. Results suggest that the application of nSi 200 mg·L^−1^ effectively increased the leaf RWC of moderately stressed plants compared to the control for both seasons.

#### 2.5.2. Electrolyte Leakage

Like leaf proline and soluble sugar contents, the electrolyte leakage generally increased with the severity of drought, but gradually decreased with increased concentrations of nSi (Figure 3b). The most pronounced effect was recorded for trees sprayed with 200 mg·L^−1^ nSi at 100% IWR, followed by those at 90% IWR that were significantly different from non-stressed trees that received 150 mg·L^−1^ nSi during the second season only. Trees sprayed with 200 mg·L^−1^ nSi showed a significant reduction in electrolyte leakage compared to those that received distilled water for both seasons, which may suggest a cumulative nSi role.

### 2.6. Oxidative Stress Markers

#### 2.6.1. Malondialdehyde (MDA)

**Figure 4:**
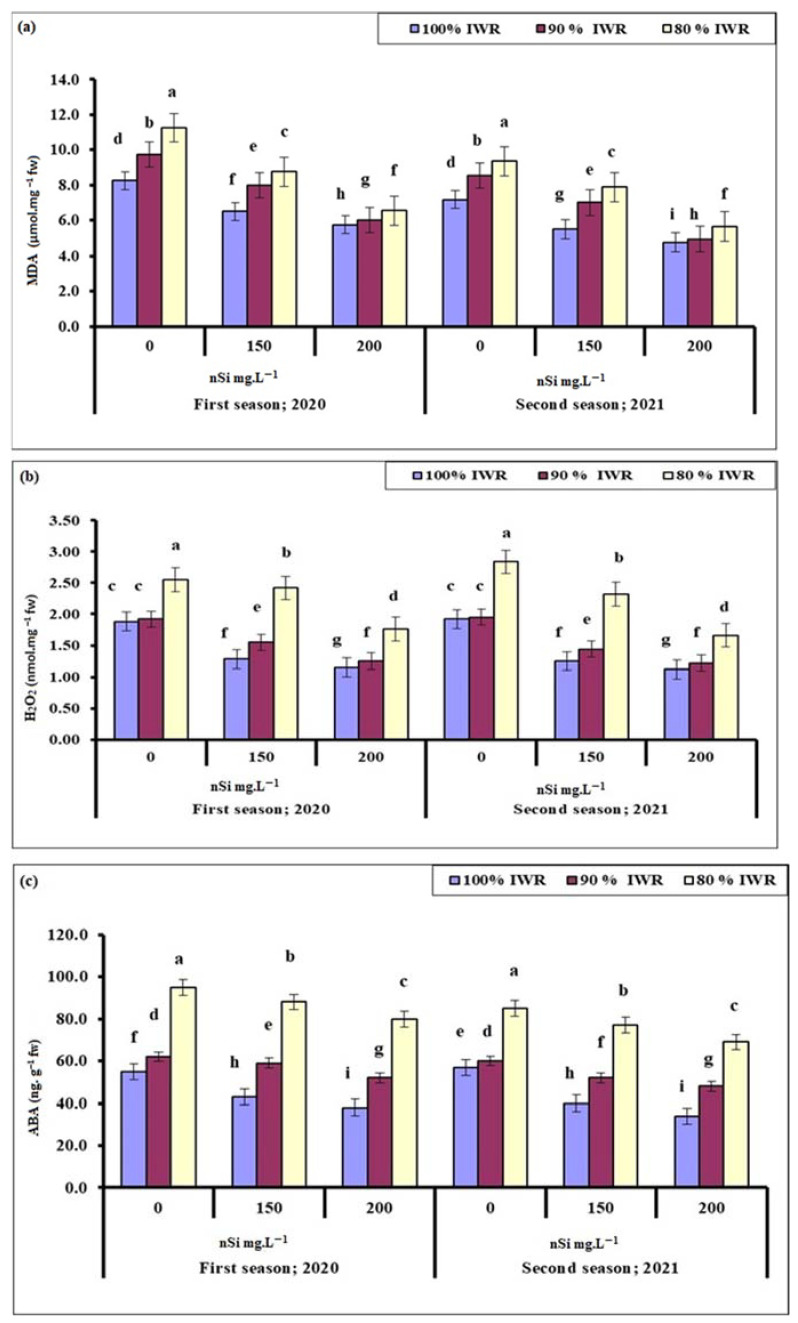
Effect of foliar sprayed nano-silicon (nSi) under different irrigation regimes on the leaf malondialdehyde ‘MDA’ (**a**), H_2_O_2_ (**b**), and abscisic acid ‘ABA’ (**c**) contents of ‘Kalamata’ olive trees during the 2020 and 2021 seasons (*n* = 10). Means with similar letters for each season are not significantly different, using Duncan’s multiple range test (DMRT) at *p* ≤ 0.05. Error bars represent the standard error of the means.

The higher the plant stress level was, the higher the oxidative stress that the plant faced, represented by the increased levels of lipid peroxidation and the production of MDA, which significantly reduced with the increased nSi concentration (Figure 4a). With regards to the stressed olive trees, the most pronounced reduction in MDA was recorded for trees that received nSi (200 mg·L^−1^) at 90% IWR, followed by those at 80% IWR during both seasons.

#### 2.6.2. Hydrogen Peroxide (H_2_O_2_)

As another indicator of plant oxidative stress, H_2_O_2_ level increased significantly with drought severity, but the application of nSi significantly decreased its levels compared to the control, with the most conspicuous effect recorded at the highest nSi concentration (200 mg·L^−1^), followed by those that received 150 mg·L^−1^, for trees subjected to 90% and 80% IWR during both seasons (Figure 4b). Trees sprayed with nSi (200 mg·L^−1^) at 80% IWR had higher H_2_O_2_ than those at 90 and 100% IWR during both seasons, suggesting that irrigation at 80% IWR was really stressful for olive trees.

#### 2.6.3. Abscisic Acid (ABA)

The non-enzymatic antioxidant, ABA (Figure 4c), showed the same response as the organic osmolytes, (e.g., proline, soluble sugars) (Figure 2b,c) to drought and nSi application during both seasons. Plant response to stress, in terms of ABA biosynthesis, matched the oxidative stress markers (MDA, H_2_O_2_) (Figure 4a,b) produced during both seasons. Likewise, in other parameters, low ABA levels indicate less stressed trees. Thus, the most effective treatments were the application of 200 mg·L^−1^, followed by 150 mg·L^−1^ nSi at 90% IWR, and then 200 mg·L^−1^, followed by 150 mg·L^−1^ nSi at 80% IWR, suggesting that 80% IWR was really stressful for olive trees under semi-arid conditions during both seasons.

## 3. Discussion

Silicon nanoparticles have been found to play an important role in a plant’s ability to overcome the adverse effects of environmental stresses [12,69,70,71,72]. Several researchers have reported the ability of nSi to improve a plant’s resistance to drought stress [29,73]. However, this is the first time nSi has been sprayed on a mature woody plant to overcome water stress. Other research work utilized nSiO_2_ to mitigate the drought effects on hawthorn (*Crataegus monogyna*) seedlings [74], and Mahaleb cherry [48].

The increase in olive yield in response to water stress was not expected (Figure 1) and contradicts the previous findings that indicated a negative effect on olive tree acclimation, and thereby yield, fruit dry mass, and oil content [75]. The total yield was also influenced by the percentage of fruit drop, which increased with the increased levels of water stress. Foliar sprayed nSi decreased the percentage of fruit drop, and lower percentages were achieved with the increase in nSi concentration (Figure 1). Trees’ response to nSi (150 mg·L^−1^), in terms of yield, varied from one season to another, while those that received 200 mg·L^−1^ showed a consistent response between seasons with the highest yield recorded for non-stressed trees, followed by those that received 90% IWR, and then trees at 80% IWR (Figure 1). Trees sprayed with nSi showed higher yield values compared to the non-treated stressed ones and the control. Foliar application of Si at tillering and anthesis stages increased the grain yield of the stressed and non-stressed plants [76]. The reduction in fruit weight, associated with increased fruit drop in stressed trees, resulted in reduced total yield (Figure 1), and was reported to make some improvement in fruit composition and oil quality [77,78,79,80]. However, average fruit weight was higher in nSi-treated trees (200 mg·L^−1^) whether stressed or not during the 2020 season, while the treated trees at reduced water levels produced heavier fruit than the non-treated stressed ones during the 2021 season, suggesting that there is a positive role of nSi in combination with normal irrigation.

Water stress reduced the leaf’s relative water content (RWC) in both years, but the application of nSi improved the RWC exponentially with increased concentration. Similar results were reported on faba beans [53], wheat [70], plums [81], and olives [13,82]. Increased level of ABA-induced stomatal closure had eventually increased RWC [83]. Chlorophyll content was the best at 200 mg·L^−1^ nSi for both stressed (90% IWR) and non-stressed trees during the 2021 season (Figure 2). Similar results were reported in faba bean, albeit a reduction in chlorophyll content was noticed with drought conditions [52]. Ma et al. [84] also reported that chlorophyll content in cucumber leaves decreased at moderate and severe drought stress. This effect has been mitigated with Si spray, which contributed to the protection of the chloroplasts. The variation in total chlorophyll content among the stressed faba bean [52], cucumber [83], and olive plants (Figure 2) could be attributed to differences in species, genotype-specific traits, growth stage, the method and concentration of nSi application, stress intensity and duration, and other environmental conditions [85]. The difference in chlorophyll content from one season to another could be due to the pre-exposure to stress that eventually improved the plant’s acclimation [86].

Photosynthesis is needed for biomass productivity. Thus, increased chlorophyll content should also increase the photosynthetic capacity, as measured by leaf total soluble sugars [87], which unexpectedly decreased. However, chlorophyll contents increased with an increase in the levels of nSi and drought (Figure 2), perhaps due to an increase in leaf RWC (Figure 3). Iron oxide nanoparticles resulted in accumulated soluble sugar contents when plants were drought-stressed. However, iron also stimulates the redox process and chlorophyll biosynthesis in plants, thus should have helped to increase leaf chlorophyll and soluble sugar contents [88,89]. Similar results were reported in stressed cotton plants, but the used nSi concentration that resulted in the highest soluble sugar contents was 3200 mg·L^−1^ [90]; about 16-fold of what has been used in the present study.

Drought stress in olives is often associated with increased cellular levels of oxidative stress markers of lipids such as MDA and H_2_O_2_ [6]. When an imbalance between reactive oxygen species (ROS) production and the antioxidant defense system occurs, increased cellular membrane damage and electrolyte leakage occur [53,83]. It was also reported that when drought-stressed olive trees were re-watered, they still exhibited higher levels of H_2_O_2_, suggesting that this drought/wet rhythm is a possible means to keep the plant’s antioxidative system on alert. It was reported that the enhanced level of H_2_O_2_ in drought-stressed leaves was accompanied by enhanced levels of MDA and electrolyte leakage [56]. In the current study, the content of H_2_O_2_ increased massively only when trees were subjected to 80% IWR (Figure 4), indicating that 90% IWR was mild water stress for olives, and the antioxidant defense system is little affected [91] since Kalamata olive is generally a drought-tolerant plant [5]. Trees sprayed with high nSi (200 mg·L^−1^) had lower leaf H_2_O_2_ when subjected to 80% IWR level (Figure 4), suggesting that the higher concentration of nSi was better at alleviating the H_2_O_2_-plant response to drought. Similar results were reported with soil application of nSi on drought-stressed barley seedlings [92]. In the current study, the electrolyte leakage (Figure 3) and MDA levels (Figure 4) also increased in response to water stress but decreased with increased nSi concentrations, which confirms the previous findings [93,94].

Proline has been described as an osmoprotectant that accumulates in response to abiotic stresses [95] and plays an essential role in the defense mechanisms of stressed plants through changes in key anatomical features of roots and leaves, the osmotic regulation of the cell sap, membrane and protein stability, enhanced enzyme activity, and scavenging the free radicals [96,97,98]. Enhanced endogenous proline levels improved leaf chlorophyll content, yield, fruit weight and diameter, and total soluble sugars (TSS) of non-stressed pomegranate [99] and orange [100], as well as salt-stressed mango [34] and tomato plants [101]. Leaf proline concentration increased with the severity of drought stress but decreased with elevated nSi concentrations (Figure 2). Increased levels of water deficit improved the biosynthesis of proline in castor bean, while foliar sprayed chitosan nanoparticles had no effect [102]. Additionally, higher concentrations of nFe mitigated the stress effects and reduced the accumulation of proline in drought-stressed wheat plants [88]. The difference in proline accumulation levels in the current study (Figure 2), compared to the previous findings could be due to the difference in water stress levels, as well as differences between wheat as an herbaceous plant and olive as a woody plant, as previously indicated [85]. However, the current results in Figure 2 contradict the previously reported findings on drought-stressed faba bean [30] and wheat [73].

The concentration of ABA increased with increased water stress levels, but decreased with increased concentration of nSi in both seasons (Figure 4), confirming the previous findings on wheat [103]. It was reported that the combined application of Si, B, Zn, and zeolite nanoparticles decreased the production of ABA in tomatoes under drought conditions [104]. Drought often leads to the formation of ROS, (e.g., H_2_O_2_, superoxide radical [O^.–^_2_], singlet oxygen [^1^O_2_], and hydroxyl radicals [OH]), which are highly toxic and can react with proteins, lipids, and DNA, accelerating the aging process of chloroplasts, thus reducing photosynthetic capacity and decreasing plant growth and productivity [105]. Abiotic environmental stresses affect the plant through osmotic stress. Cell homeostasis is maintained against osmotic stress by the mechanism of osmotic adjustment, which is a primary stress-adaptive motor that positively correlates with plant production under drought conditions in various crops [106]. The mechanism of osmotic adjustment leads to the synthesis of organic osmolytes, (e.g., sugars, proline) [107], non-enzymatic antioxidants, (e.g., ascorbate, glutathione), and enzymatic (scavenger enzymes) antioxidants, (e.g., SOD, CAT, POD) [108] to balance the osmotic pressure of the cytosol and vacuole with that of the external environment [109]. The biosynthesis of ABA usually occurs in the roots under drought conditions and can increase up to 50 times, which is considered the highest change in any phytohormones under such conditions [110]. The ABA concentration has been shown to promote root growth and adjust shoot growth [83,111] via the regulation of transpiration and photosynthesis, as well as its potent effect on the production of primary and secondary metabolites, antioxidant enzymes, and lipoxygenase inhibitory activity [112,113,114]. A cross-talk mechanism between Ca^+2^ and ROS that originates from NADPH-oxidase in the ABA-induced antioxidant defense in the plant was also reported [115]. The role of ABA on sugar contents came through the accumulation of assimilates from the phloem into the fruit by strengthening sink capacity [116,117]. On the other hand, an increase in ABA has been reported as an indicator of the beginning of fruit senescence [118]. It was reported that the increased levels of ABA under drought conditions may trigger ethylene production, which promotes the activity of hydrolytic enzymes, such as endo-beta-glucan, cellulase, and polygalacturonase at the abscission zone of the fruit petiole inducing preharvest fruit abscission [119,120,121].

Overall, it could be said that the foliar application of nSi at 200 mg·L^−1^ followed by 150 mg·L^−1^ was effective in alleviating moderate drought effects (90% IWR) in ‘Kalamata’ olive trees compared to severe drought (80% IWR). During severe drought at 80% IWR, the tree’s yield and average fruit weight reduced whilst fruit drop, along with levels of osmoprotectants increased with more membrane damage. The results of this study suggest that ‘Kalamata’ olive trees were severely stressed at 80% IWR compared to 90% IWR reinforcing its classification as drought sensitive [4] compared to most olive cultivars that are classified as drought tolerant [5].

## 4. Materials and Methods

### 4.1. Experiment

This research was carried out on nine-year-old ‘Kalamata’ olive trees (*Olea europaea* L.) grown in sandy soil in a private orchard located at Wadi El-Natrun, Beheira Governorate (30°46′98″ N, 30°27′43″ E, 23 m below the Mediterranean Sea level and 38 m below the Nile River), Egypt, during the 2020 and 2021 seasons. Ninety trees planted at a 6 m × 6 m spacing, similar in vigor and size, free from any symptoms of physiological disorders or nutrient deficiencies, were chosen for this experiment. Trees were subjected to drip irrigation from deep groundwater well and received other agricultural practices as the entire orchard during both seasons. Soil and water analysis were performed according to the methods described by Wilde et al. [122] and displayed in Table 1.

Irrigation treatments started in spring by full bloom (≈mid-March) and ceased at harvest by fall (≈late October). Trees were subjected to deficit irrigation based on differences in crop evapotranspiration (*ETc*), and three irrigation treatments–100% (control), 90%, and 80%–were used. The percentage of *ETc* was calculated based on the reference crop evapotranspiration (*ETo*) [mm·day^−1^] that is presented in Table 2 and crop coefficient factor (*K_c_*) of olive, as suggested [123], using the following equations:

*ETc* = *ETo* × *K_c_* (1)

Irrigation water requirements (IWR) for each irrigation regime (m^3^·ha^−1^·season^−1^) during the entire season were determined using the following equation:

*IWR* = (*A* × *ETc* × *Ii* × *Kr*)/(*Ea* × 1000 (1 − *LR*)) (2)

where *ETC* expressed as m^3^·ha^−1^ per irrigation time, *A* = cultivated area (ha), *ETc* = crop evapotranspiration, *Ii* = irrigation interval (day), *Kr* = reduction factor, *Ea* = irrigation efficiency, *LR* = leaching requirement = 10% of the total water amount delivered to the treatment.

The reduction factor was determined by the following equation:

*Kr* = (0.10 + *GC*) ≤ 1 (3)

where *GC* = the ground cover.

The leaching requirements (*LR*) were estimated according to the following equation:

*LR* = *ECw*/2*ECe _max_* (4)

where *ECw* = the electrical conductivity of the irrigation water (dS·m^−1^), 2*ECe _max_* = the maximum electrical conductivity of the soil saturated extract for a given crop.

The number of irrigation times varied among the three treatments with a frequency of 1–5 irrigation times per week, based on weather conditions (Table 2) and soil water content that was monitored weekly using soil tensiometer Model 64xx series (Spectrum Technologies Inc., Aurora, IL, USA). Two lateral lines of irrigation pipes (one on each side of the trees row) with 10 drippers per tree (8 L·h^−1^·dripper^−1^) were used for the control treatment (100% IWR), whereas 9 and 8 drippers were used for the 90% and 80% IWR treatments, which represent a total of 7007.14, 6306.43 and 5605.71 m^3^·ha^−1^·season^−1^, respectively.

Foliar spray with nanoparticle chelate fertilizer of silicon (nSi = 5–15 nm) (Sigma-Aldrich, St. Louis, MO, USA) at 150 and 200 mg·L^−1^, supplemented with Tween 20 as a surfactant (Sigma-Aldrich, St. Louis, MO, USA), was applied three times; by the onset of the vegetative growth in spring (≈late February), at full bloom (≈mid-March), and at fruit set (≈early April). Control trees were also sprayed at the same times with distilled water, supplemented with Tween 20 to avoid any effects between the treatments and the control, which could be related to the surfactant. Every tree received about 15 liters of the spray solution until dripping during the early morning period.

### 4.2. Yield and Average Fruit Weight

Total yield (kg·tree^−1^) was recorded by harvest time in late October, and average fruit weight (g) was determined by weighing 90 randomly selected fruit samples from each replicate using a bench-top digital scale Model PC-500 (Doran scales, Inc., Batavia, IL, USA).

### 4.3. Fruit Drop

Fruit drop percentage was estimated per tree by randomly selecting four branches from the four directions (N, E, S, and W), and then branches were wrapped using net bags, and dropped fruit were collected and counted every 15 days until harvest. The number of the remaining fruit on the branches was recorded by the last observation time. The fruit drop percentage was calculated using the following equation:

Fruit drop (%) = [(Initial fruit number − Final fruit number)/Initial fruit number] × 100(5)

### 4.4. Leaf Analysis

By the end of each harvest season, a sample of 50 mid-branch leaves was randomly collected, from the four directions (N, E, S, and W) and three levels (top, medium, and bottom) of the tree, for leaf analysis. All used chemicals were imported from Sigma-Aldrich, St. Louis, MO, USA.

Leaf chloroplasts were extracted in 85% acetone solution, and the absorbance of the aqueous phase of the extracted solution was estimated using a spectrophotometer Model UV-120-20 (Shimadzu, Kyoto, Japan) at λ = 663 and 645 nm [124]. Total chlorophyll was then calculated using the following equation:

Total chlorophyll (mg·100 g^−1^ fw) = [(20.2 × OD 645 nm + 8.02 × OD 663 nm) × V]/(fw × 1000) (6)

where: OD = optical density, V = the final volume of the solution (mL), and fw = tissue fresh weight (g).

Leaf proline (mg·100 g^−1^ fw) was extracted using 0.5 g of young leaves with sulfuric acid (3%), and the solution was quantified using ninhydrin reagent [125]. The solution was then mixed with toluene, and the absorbance of the toluene phase of the extracted solution was determined using the spectrophotometer at 520 nm.

The concentration of the soluble sugars (mg·100 g^−1^ fw) was determined using dried leaf samples [126]. Every dried leaf sample (150 mg) was extracted twice with 80% ethanol and centrifuged at 3500 rpm for 10 min and the volume of the supernatant was adjusted to 25 mL. The supernatant (1 mL) was then transferred to a test tube with the addition of 1 mL phenol (18%) and 5 mL sulfuric acid, and the mixture was shaken. The absorbance of the aqueous phase of the extracted solution was recorded at 490 nm using the spectrophotometer.

The RWC of the leaf was estimated using a fresh leaf sample (0.2 g) incubated in distilled water (50 mL) for 4 h. The turgid weight of the leaf sample was calculated, and then the sample was oven-dried at 60 °C for 48 h, followed by the determination of the dry weight [124]. Leaf RWC was calculated using the following equation:

RWC (%) = [(FW − DW)/(TW − DW)] × 100 (7)

where FW, DW, and TW = fresh, dry, and turgid weights, respectively.

Ten discs per leaf (0.5 cm diameter) were collected from ten freshly expanded leaves and used to determine the electrolyte leakage of the membrane [124]. Leaf discs were washed three times with deionized water to remove dust, and then kept in closed tubes containing 10 mL of deionized water and shook for 30 min using a lab shaker, Model Bioshake 3000-T (Kobenhavn, NV, Danmark), and left in a dark at room temperature (≈22–23 °C) for 24 h. The initial electrical conductivity of the solution (EC1) was determined using an electrical conductivity meter, Model HI9032 (Hanna Instruments, Woonsocket, RI, USA). Samples were then kept in a ‘Precision ^TM^ General Purpose’ water bath (ThermoFisher Scientific, Waltham, MA, USA) at 80 °C for 20 min to release all endogenous electrolytes. Afterward, the solution was cooled down to 25 °C, its final electrical conductivity (EC2) was estimated, and the percentage of EL was calculated using the following equation:

EL (%) = (EC1/EC2) × 100 (8)

Lipid peroxidation of the membrane was determined with MDA concentration (μmol·mg^−1^ fw) using the thiobarbituric acid reactive substance assay (TBARS) [127]. A fresh leaf sample (100 mg) was extracted in 1% trichloroacetic acid (TCA) and then centrifuged for 10 min at 10,000× *g* using a benchtop general purpose centrifuge Model Allegra V-15R (Beckman Coulter Life Sciences, Indianapolis, IN, USA). The supernatant (1 mL) was mixed with 4 mL Thiobarbituric acid (TBA) [0.5%], heated for 30 min at 95 °C, and then cooled in an ice bath, followed by centrifugation at 5000× *g* for 5 min. The absorbance of the aqueous phase of the extracted solution was recorded at 532 and 600 nm using the spectrophotometer.

The non-radical H_2_O_2_ (nmol·g^−1^ fw) was determined by homogenizing a fresh leaf sample (100 mg) in 0.1% trichloroacetic acid (TCA). The homogenate was centrifuged at 12,000× *g* for 15 min, and a sample of the supernatant (0.5 mL) was mixed with 0.5 mL potassium phosphate buffer (10 mM, pH 7.0) and 1 mL potassium iodide (1 M). The absorbance of the aqueous phase of the extracted solution was recorded at 390 nm using the spectrophotometer, and a standard curve was used to calculate H_2_O_2_ content [128].

Leaf ABA content (ng·g^−1^ fw) was determined according to Koshioka et al. [129] using high-performance liquid chromatography (M5 Microflow HPLC system; SCIEX, Framingham, MA, USA).

### 4.5. Experimental Design and Statistical Analysis

The experimental design was in a randomized complete block system, as a split-plot experiment of three nano-silicon concentrations (main plots) and three irrigation regimes (sub-plots); a total of nine treatments, 10 replicates each. Each replicate was represented by one tree [130].

Data were analyzed using CoStat—Statistics Software (version 4.20) [131]. Data were first run for numerical normality and homogeneity of variance using the Shapiro–Wilk’s and Levene’s tests, respectively, and then the analysis of variance was performed, and means were compared using Duncan’s multiple range tests (DMRT) at *p* ≤ 0.05. Standard error bars were also added for mean comparisons in the figures [132,133].

## 5. Conclusions

Imposing water stress on a drought-sensitive ‘Kalamata’ olive trees induced oxidative stress, which was expressed as elevated H_2_O_2_, MDA, and electrolyte leakage with an increased fruit drop. The application of nSi generally improved fruit yield, fruit weight, leaf total chlorophyll, and RWC, and lowered fruit drop, leaf proline, soluble sugars, H_2_O_2_, electrolyte leakage, and ABA, with a more pronounced effect at moderate water stress (90% IWR). Increasing the nSi foliar concentration from 150 to 200 mg·L^−1^ improved the morphological, physiological, and biochemical characteristics of the olive trees, resulting in improved growth, development, and productivity under semi-arid conditions. Future research could study the molecular basis of olive defense mechanisms to enhance the drought tolerance of the ‘Kalamata’ cultivar in order to withstand more severe drought conditions, like other olive cultivars, amid the global water scarcity. Future research could also explore the cold tolerance mechanisms of this cultivar.

## Figures and Tables

**Table 1:** Soil and water analysis of the experimental site.

	Soil (0–40 cm)	Water
pH	8.22	7.01
Sand (%)	92.0	–
Silt (%)	5.0	–
Clay (%)	3.0	–
EC (dS/m)	1.82	1.56
CaCO_3_ (%)	3.4	–
Ca^2+^ (meq·100 g^−1^)	8.6	9.4
Mg^2+^ (meq·100 g^−1^)	3.2	4.3
Na^+^ (meq·100 g^−1^)	6.9	9.80
K^+^ (meq·100 g^−1^)	1.5	0.22
Cl^−^ (meq·100 g^−1^)	8.2	6.46
SO_4_^2–^ (meq·100 g^−1^)	6.4	14.3
CO^3–^ (meq·100 g^−1^)	0.0	–
HCO^3–^ (meq·100 g^−1^)	5.6	3.0

**Table 2:** Average meteorological data of Wadi El Natrun area (2020 and 2021), source: own elaboration.

	Jan	Feb	Mar	Apr	May	Jun	Jul	Aug	Sept	Oct	Nov	Dec
Temp. mean Max (°C)	20.7	25.5	25.7	27.1	32.9	33.8	34.8	34.9	32.8	28	23.2	20.7
Temp. mean Min (°C)	9.1	8.98	11.1	13.7	16.7	19.5	20.2	22.7	20.2	17	10	9.3
Temp. average (°C)	14.9	17.24	19.4	20.4	24.3	26.65	27.45	28.5	26.4	23.4	20.05	15
Relative humidity (%)	65.1	62.5	62.56	58	58.1	59.2	58.8	59.9	63.1	62	65.1	65.2
Evaporation (mm·day^−1^)	6.2	7.7	9.8	12.5	13.8	15	14.3	12.7	10.5	8.6	6.1	5.1
*ETo* (mm·day^−1^)	2.80	3.30	4.0	4.80	5.30	5.80	6.10	5.40	4.40	3.10	3.02	2.90

## Data Availability

Not applicable.
